# Left ventricular concentric geometry predicts incident diabetes mellitus independent of established risk factors in the general population: the Copenhagen City Heart Study

**DOI:** 10.1186/s12933-019-0842-0

**Published:** 2019-03-20

**Authors:** Daniel Modin, Rasmus Møgelvang, Peter Godsk Jørgensen, Magnus Thorsten Jensen, Jelena P. Seferovic, Tor Biering-Sørensen

**Affiliations:** 1 Cardiovascular Non-Invasive Imaging Research Laboratory, Department of Cardiology, Herlev & Gentofte Hospital, University of Copenhagen, Copenhagen, Denmark; 2 Institute of Clinical Medicine, Faculty of Health Sciences, University of Copenhagen, Copenhagen, Denmark; 3 Clinic of Endocrinology, Diabetes and Metabolic Disorders, Clinical Center of Serbia, Belgrade, Serbia; 4 Department of Biomedical Sciences, Faculty of Health and Medical Sciences, University of Copenhagen, Copenhagen, Denmark

## Abstract

**Background:**

Subtle impairments in left ventricular (LV) function and geometry are common findings in individuals with diabetes. However, whether these impairments precede the development of diabetes mellitus (DM) is not entirely clear.

**Methods:**

Echocardiograms from 1710 individuals from the general population free of prevalent diabetes mellitus were analyzed. Left ventricular (LV) concentric geometry was defined as either LV concentric remodeling or LV concentric hypertrophy as directed in contemporary guidelines. The severity of LV concentricity was assessed by relative wall thickness (RWT) calculated as posterior wall thickness (PWT) indexed to left ventricular internal diameter at end diastole (LVIDd) (RWT = 2 * PWT/LVIDd). End-point was incident DM.

**Results:**

Median follow-up time was 12.6 years (IQR: 12.0–12.8 years). Follow-up was a 100%. A total of 55 participants (3.3%) developed DM during follow-up. At baseline, the prevalence of a concentric LV geometric pattern was significantly higher (41.8% vs 20.3%, p < 0.001) in individuals who developed DM during follow-up. In a final multivariable model adjusting for established DM risk factors including HbA1c, BMI and plasma glucose, LV concentric geometry and RWT remained significantly associated with incident DM (LV concentric geometry: HR 1.99, 95% CI 1.11–3.57, p = 0.021) (RWT: HR 1.41, 95% CI 1.06–1.86, p = 0.017, per 0.1 increase). This association remained despite adjustment for established risk factors for DM.

**Conclusion:**

Altered LV geometry may precede the development of DM. LV concentric geometry determined by echocardiography and the severity of LV concentricity evaluated as RWT are associated with incident DM in the general population.

**Electronic supplementary material:**

The online version of this article (10.1186/s12933-019-0842-0) contains supplementary material, which is available to authorized users.

## Introduction

Diabetes mellitus (DM) is a major risk factor for cardiovascular disease (CVD) [1], and the increasing prevalence of DM in the years to come represents one of the greatest threats to public health. In 2012, in the US, 1 out of every 11 individuals was living with diabetes, and 1 out of every 3rd adult was living with prediabetes [2]. In 2012, this astronomical burden of disease resulted in a cost of 245 billion dollars in the US alone [2]. Since many risk factors for diabetes are modifiable, identification of individuals at high risk of developing diabetes is needed to initiate preventive measures as early as possible.

Glucose homeostasis is closely tied to cardiovascular pathology, and even in subjects not diagnosed with fulminant DM, dysfunctional glucose homeostasis significantly increases the risk of CVD [3]. Cross-sectional studies have demonstrated impairments in diastolic function and increases in left ventricular (LV) mass and wall thickness to be the first manifestations of myocardial involvement in DM [4, 5]. These changes are characteristic of the cardiac impairment observed in diabetes [6]. Specifically, a concentric LV geometric pattern quantified by relative wall thickness has been tied to DM and abnormal glucose homeostasis [7].

Recently, evidence has indicated that myocardial involvement and early diastolic dysfunction may precede the development of DM [8–10]. This suggests that indices of myocardial structure and function may offer information value in predicting DM. Only one study, published in March 2017, have investigated the prognostic value of myocardial indices derived from echocardiography with regards to the development of DM in the general population [9]. However, this study employed multivariable logistic regression and not survival analysis to assess the predictive value of echocardiography, and the general population was set in Korea [9], making the results not directly applicable to Caucasians taking into account that ethnicity in itself is a significant risk factor for DM, and that ethnicity modifies the relationship between several risk factors and DM [11, 12]. Thus, this study sought to determine the prognostic role of echocardiography in predicting incident DM in a general population of European origin. Finally, in addition to improving risk stratification, the results of this study may also help to shed more light on the conundrum that is diabetic cardiomyopathy today [13].

## Methods

### Data availability

The data, analytic methods, and study materials will not be made available to other researchers for purposes of reproducing the results or replicating the procedure. The data analysed in this paper is governed by the Danish Data Protection Agency and can only be made available for any additional researcher if a formal application is made to the Danish authorities.

### Population

**Fig. 1:**
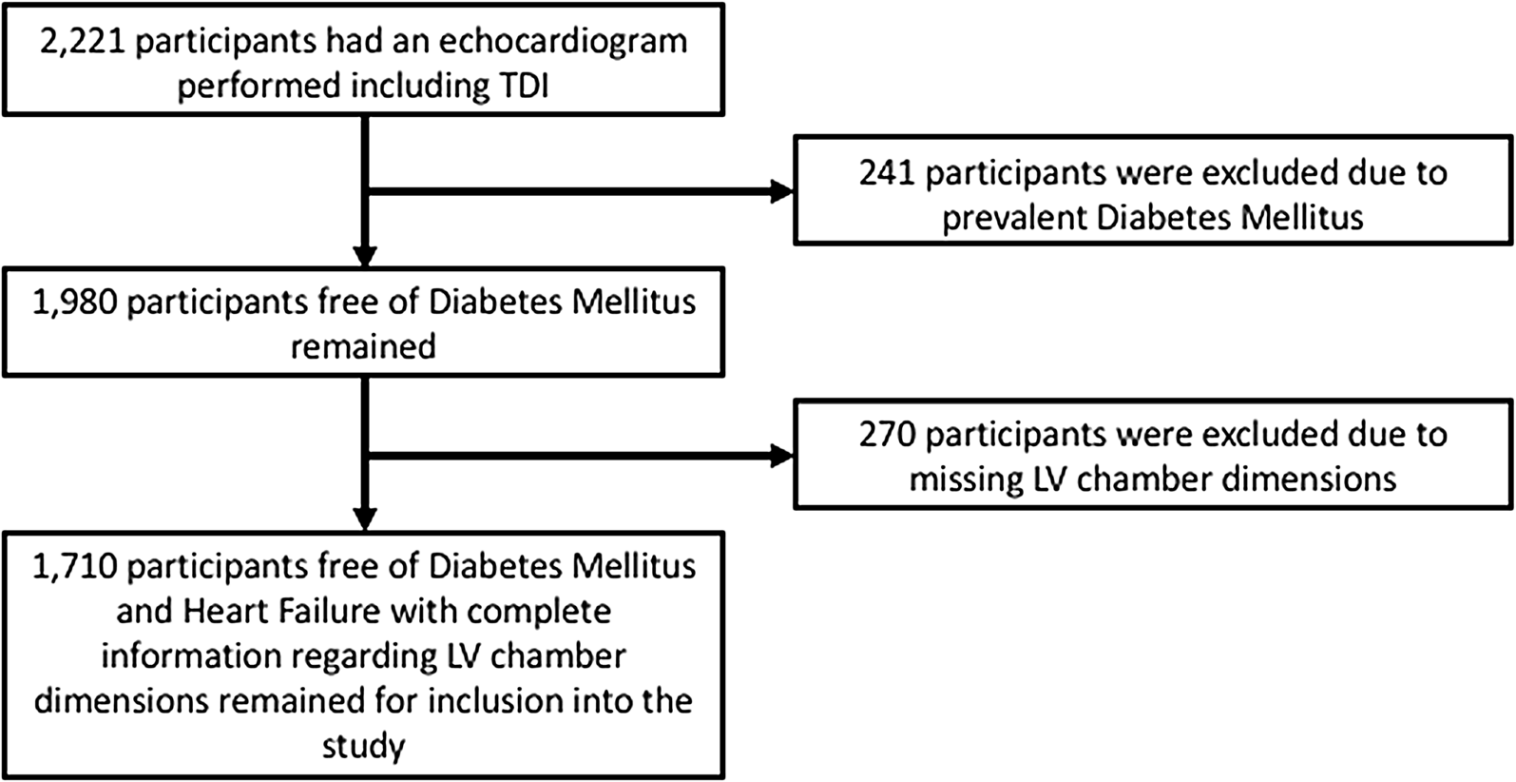
Flow diagram of the study sample selection process. *TDI* tissue Doppler imaging, *LV* left ventricle

The Copenhagen City Heart Study is a longitudinal cohort study designed to identify and characterize risk factors for CVD in the general population. The study population is based in and around the city of Copenhagen, Denmark. The population has been previously described in extensive detail [14–17]. This echocardiographic sub study included all participants who had an echocardiographic exam including tissue Doppler imaging performed in the 4th round of examination, conducted from 2001 to 2003. Whether any specific participant underwent an echocardiographic exam was independent of both health status and risk factors. Figure 1 displays the flow diagram for the study sample selection process. Initially, 2221 participants underwent echocardiography including tissue Doppler echocardiography (Fig. 1). A total of 241 participants were excluded due to prevalent diabetes (Fig. 1). Then 270 participants had to be excluded due to missing LV chamber dimensions (Fig. 1). This left 1710 participants for inclusion into the study (Fig. 1). In the final cohort, no participants had significant valvular disease.

### Ethics

Written consent was collected from all participants. The study complies with the second declaration of Helsinki. The design of the study was approved by a regional scientific ethics committee.

### Health examination

All participants underwent a general health examination consisting of a questionnaire and a physical examination. Blood pressure readings were obtained by sphygmomanometer. Plasma cholesterol and plasma glucose levels were collected using non-fasting venous blood samples [18]. Plasma-pro-BNP (pro B-type natriuretic peptide) was measured by a processing-independent assay and abnormal values defined as pro-BNP > 150 pmol/L. Prevalent DM was defined as either a plasma glucose level ≥ 11.1 mmol/L or HbA1c ≥ 7.0% or the use of glucose-lowering medication [19]. Prevalent ischemic heart disease (IHD) was defined as either a history of admission for acute coronary artery occlusion, percutaneous coronary intervention, coronary artery bypass grafting or major electrocardiogram alterations per Minnesota codes 1.1 to 3.

### Echocardiographic examination

Echocardiographic examinations were carried out by 3 experienced sonographers using Vivid 5 Ultrasound machines (GE Healthcare, Horten, Norway) with a 2.5 MHz transducer. All participants were subject to both conventional 2D-imaging and color tissue Doppler imaging (TDI). Echocardiograms were stored on magneto-optical disks and on an external FireWire harddrive (Lacie, France). All echocardiograms underwent offline analysis by experienced investigators blinded to all clinical data and information about outcomes using commercially available software (EchoPac, GE Healthcare, Horten, Norway).

### Conventional echocardiography

Wall motion score indexing (WMSI) was determined using the 16-segment model as directed by the American Society of Echocardiography [20] with subsequent estimation of the left ventricular ejection fraction (LVEF) from the WMSI-score. An M-mode still frame perpendicular to the long axis of the LV at the level of the mitral valve leaflets was used to determine LV diastolic dimensions and LV wall dimensions. Left ventricular mass index (LVMI) was derived by indexation of LV mass with body surface area (BSA). Relative wall thickness (RWT) was calculated by indexation of the posterior wall thickness (PWT) with the left ventricular internal diameter at end diastole (LVIDd) (RWT = 2 * PWT/LVIDd) [20]. LV hypertrophy categories were defined from RWT and LVMI as directed in contemporary guidelines [20]. LV concentric geometry was defined as either LV concentric remodelling (RWT > 0.42 and LVMI ≤ 115 for men or RWT > 0.42 and LVMI ≤ 95 for women) or LV concentric hypertrophy (RWT > 0.42 and LVMI ≥ 115 for men or RWT > 0.42 and LVMI ≥ 95for women [20]. Average wall thickness was defined as the sum of interventricular septum diameter (IVSD) and PWT divided by 2 ((IVSD + PWT)/2). Mitral valve inflow patterns were determined using pulsed wave Doppler in the apical position between the mitral valve leaflet tips. From here, peak early (E) and late (A) inflow velocity was measured and E/A ratio was calculated. Furthermore, the deceleration time of the E wave (DT) was measured.

### Color tissue Doppler imaging

Color TDI velocity tracings were used to determine the peak longitudinal systolic velocity (s′), the early peak longitudinal diastolic velocity (e′) and the late longitudinal peak diastolic velocity (a′). In the 4-chamber view, the TDI range gate was placed in the septal and lateral position of the mitral annulus with subsequent determination of peak velocities from the TDI velocity tracings. Thus, s′, e′ and a′ were calculated as the averages of the septal and lateral values. Aortic valve timings were determined using a 2–4 cm straight M-mode line through the septal half of the mitral leaflet in the color TDI 4-chamber view [15].

### Follow-up and outcome

Participants were followed from echocardiographic examination in 2001 to 2003 until time to event or, in the case of no event, until October 2014. The end-point of this study was incident DM. Follow-up data was collected through the Danish National Board of Health’s National Patient Registry using International Classification of Diseases, 10th revision codes (ICD). DM was defined as ICD-10 codes DE10-DE14.

### Statistics

**Fig. 2:**
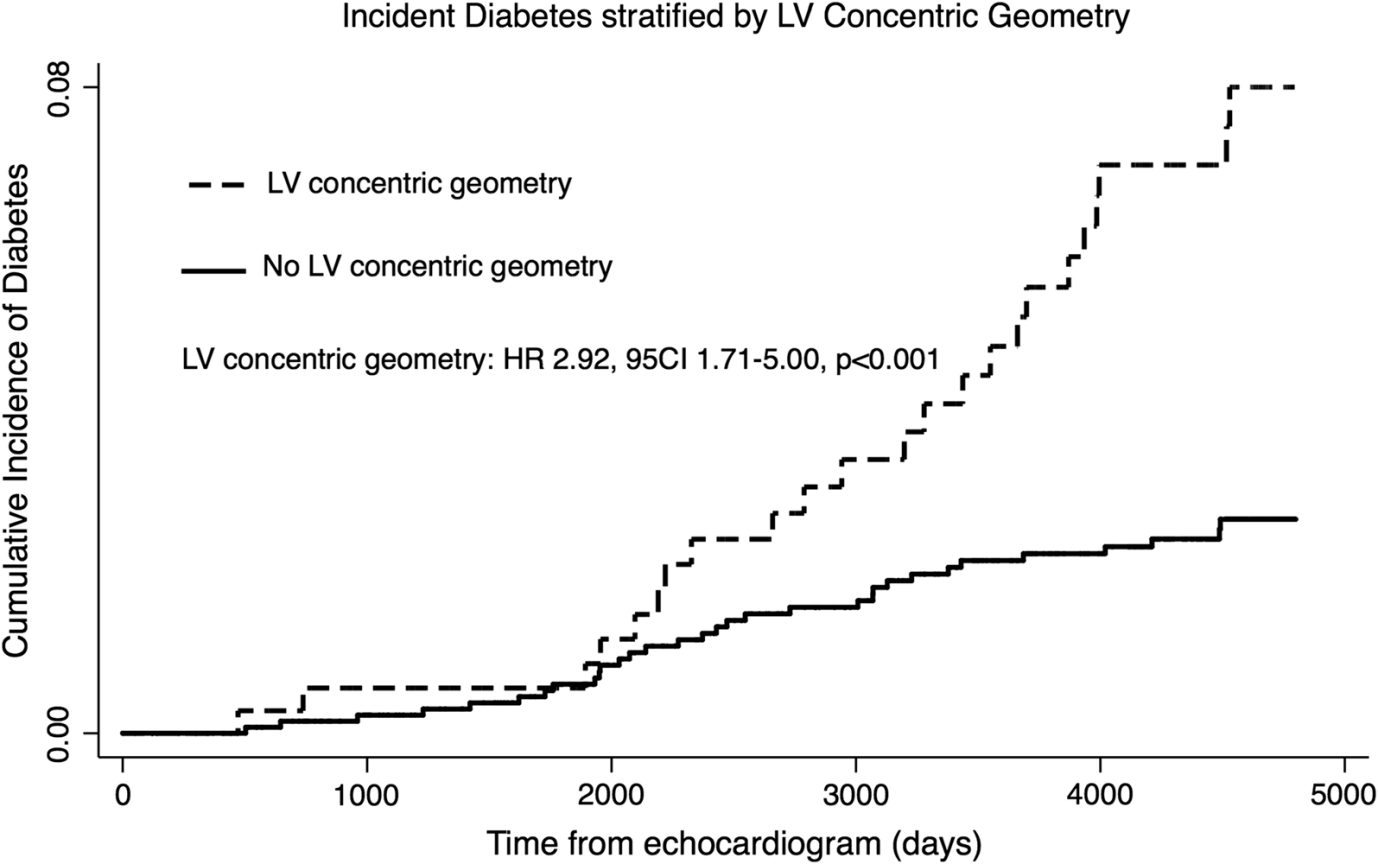
Incident diabetes mellitus stratified by left ventricular (LV) concentric geometry at baseline

**Fig. 3:**
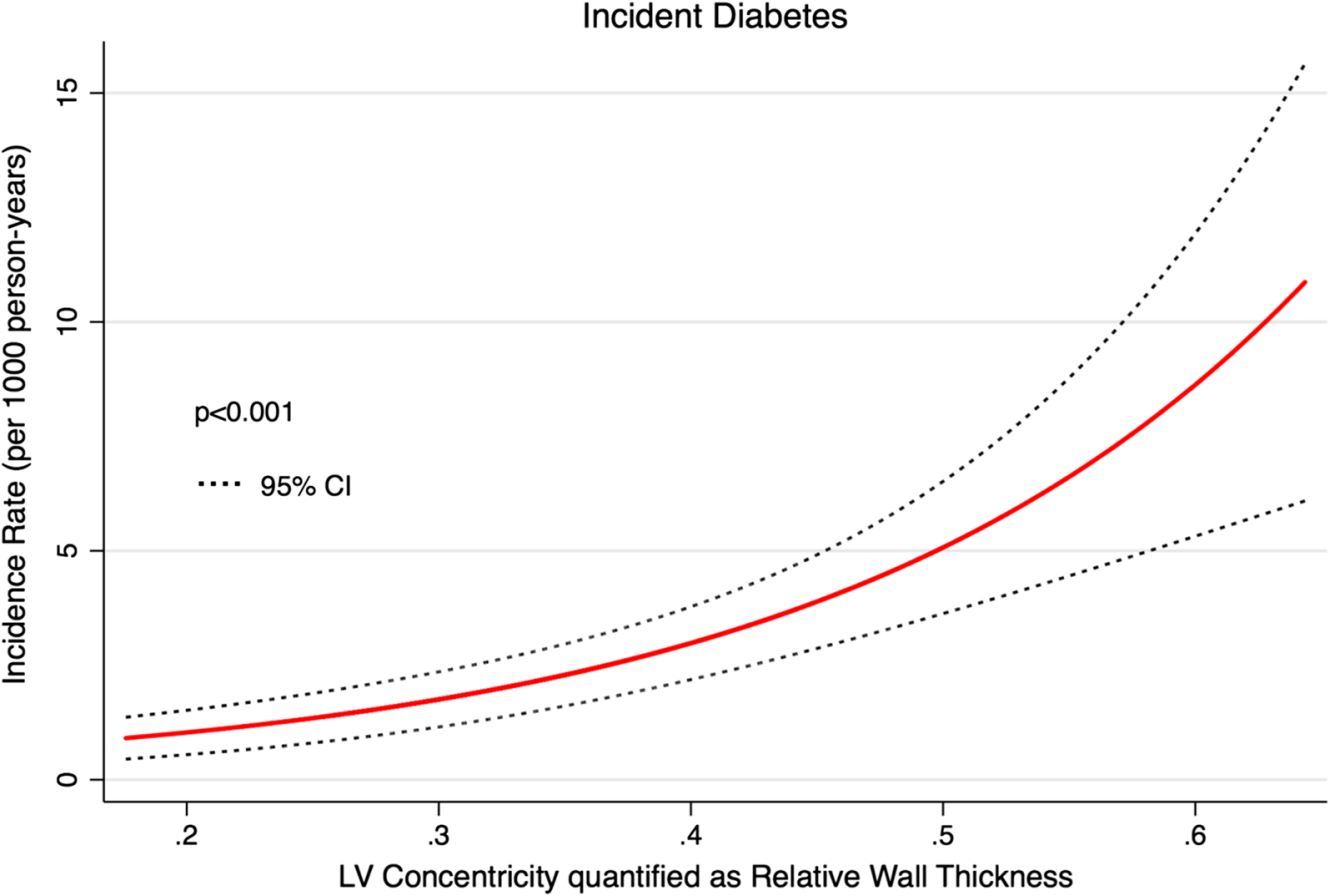
Long-term risk of incident diabetes mellitus as a function of the degree of left ventricular (LV) concentricity quantified as relative wall thickness (RWT)

**Fig. 4:**
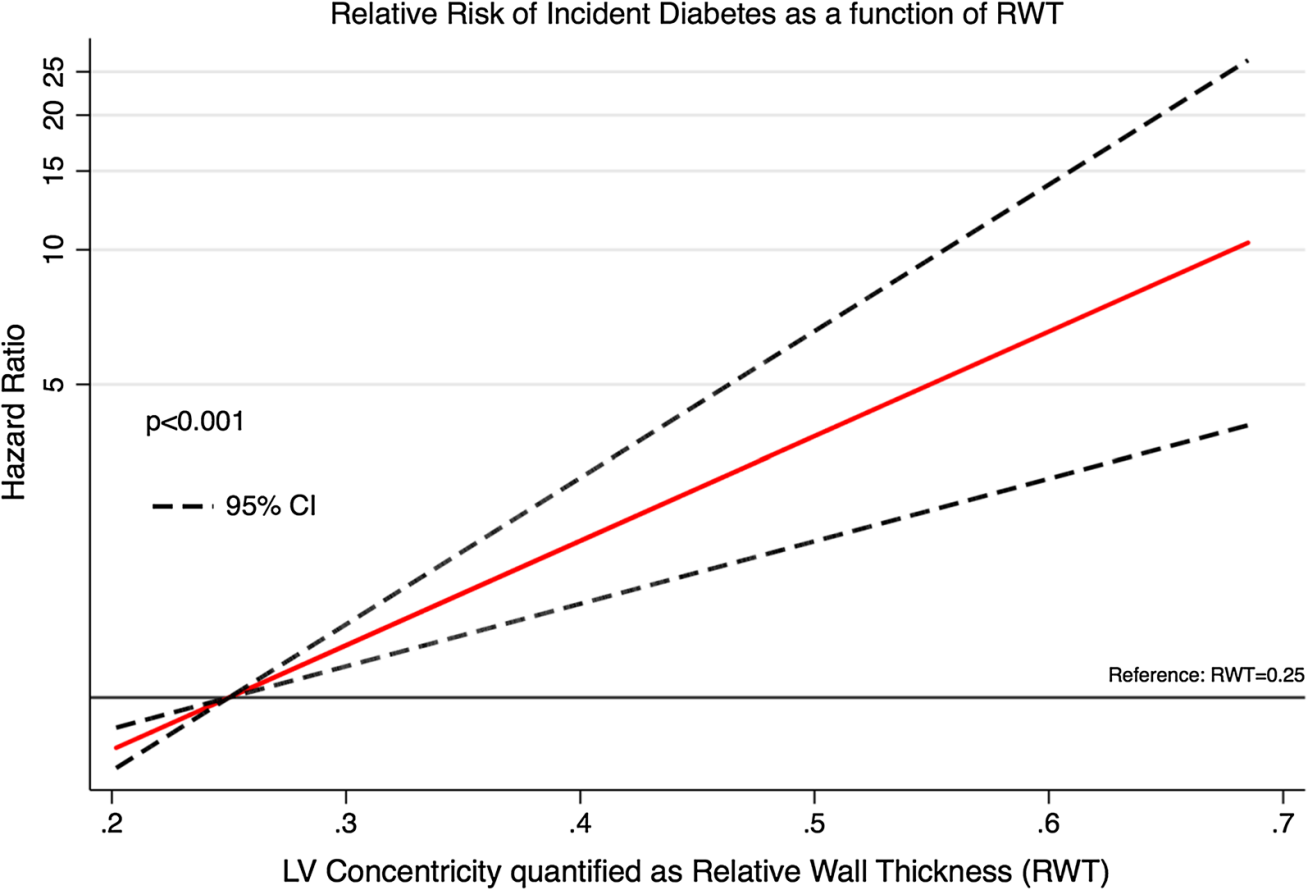
Long-term relative risk of incident diabetes mellitus as a function of the degree of left ventricular (LV) concentricity quantified as relative wall thickness (RWT). The y-axis is logarithmically scaled

**Fig. 5:**
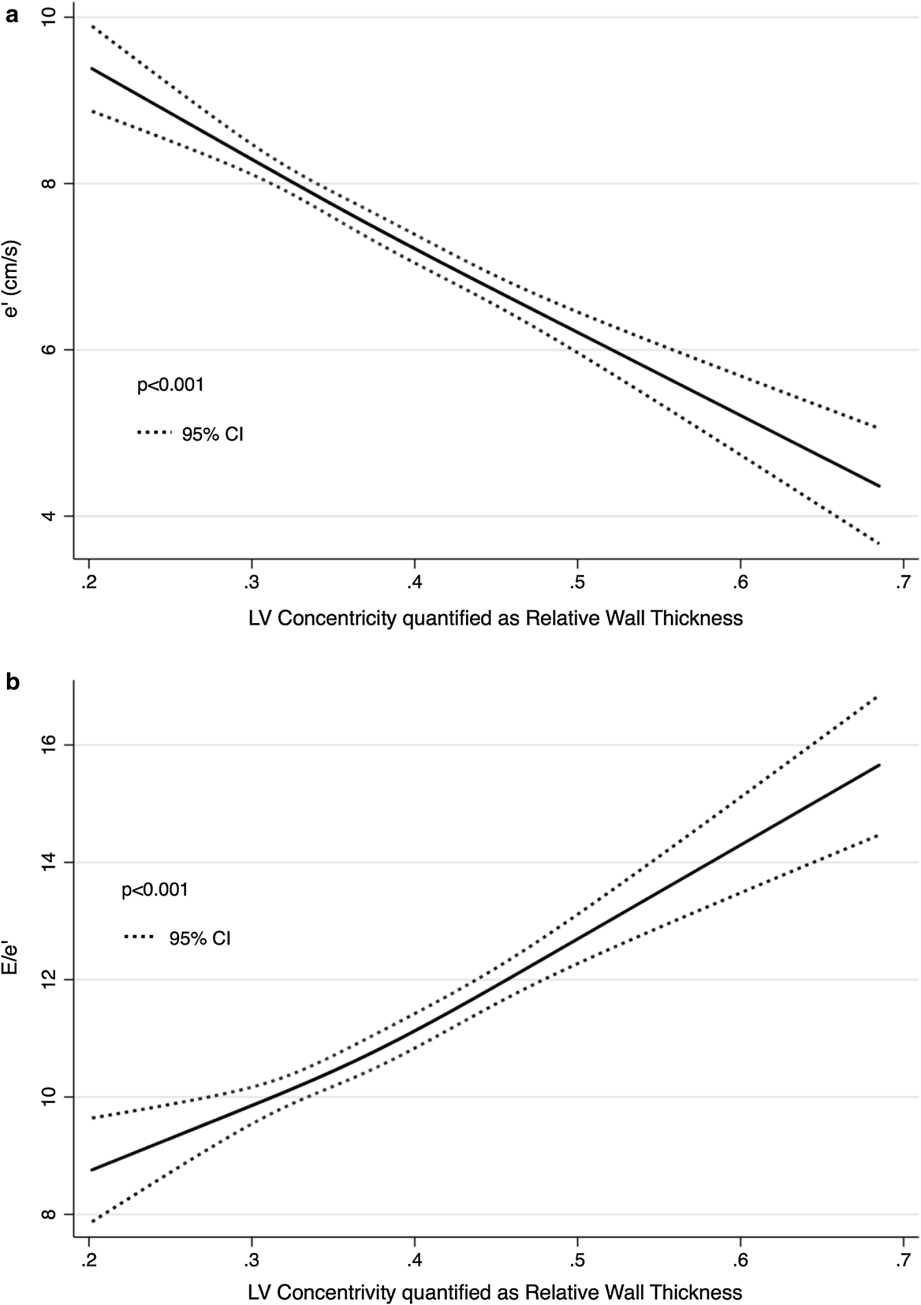
The association between LV concentricity quantified as relative wall thickness and diastolic function as determined by e′ and E/e′

All statistical analysis was done using STATA 13 for Mac OS. A p-value < 0.05 was considered statistically significant. In Table 1, continuous variables exhibiting Gaussian distribution were compared using the two-tailed students t-test. Continuous variables not displaying Gaussian distribution were compared using the Mann–Whitney U-test. Proportions were compared using the Chi squared test. In Table 2, univariable and multivariable Cox regression was used to assess prognostic strength of examined parameters. The extent of the multivariable analysis was limited by the relatively small number of events [21]. In Fig. 2, survival curves were constructed using the Kaplan–Meier method. In Fig. 3, Poisson cubic spline regression was used to estimate incidence rates of DM using RWT as a continuous measure of LV concentricity. In Fig. 4, Cox cubic spline regression was used to estimate the hazard ratio associated with increasing degree of LV concentricity evaluated as RWT. In Figs. 3 and 4 the number of knots were selected by calculating the Akaike Information Criterion (AIC) for each model with subsequent selection of the model displaying the lowest value. In Fig. 5 we studied the relationship between the degree of LV concentricity evaluated as RWT and diastolic function as quantified by E/e′ and e′ using restricted cubic spline regression. For the number of knots we chose the best fitting model as determined by the lowest AIC. We used Net Reclassification Analysis [22] and Integrated Discrimination Improvement Analysis [22] to evaluate the incremental prognostic value of LV concentric geometry and RWT in predicting DM over a model of established risk factors for DM. This model was limited by the number of events (n = 55) in the final multivariable model and hence the following risk factors were chosen: age, sex, systolic blood pressure, total cholesterol, triglycerides, HbA1c levels, and BMI. Finally, we assessed whether treating death as a competing event in competing risk Cox regressions changed our results (Additional file 1: Table S1).

**Table 1:** Baseline characteristics of participants stratified according to DM at follow-up

Demographics	All participants	No DM	DM	p-value
N	1710	1655 (96.8%)	55 (3.2%)	
Age (years)	56.8 (16.3)	56.4 (16.4)	65.5 (10.9)	< 0.001
Male	676 (39.5%)	648 (39.2%)	28 (50.9%)	0.08
Clinical characteristics				
Systolic BP (mmHg)	133.9 (22.4)	133.4 (22.4)	148.2 (18.6)	< 0.001
Diastolic BP (mmHg)	77.8 (12.0)	77.6 (12.1)	82.6 (10.3)	0.002
Hypertension	665 (39.0%)	625 (37.9%)	40 (72.7%)	< 0.001
MAP (mmHg)	96.5 (13.9)	96.2 (13.9)	104.5 (11.2)	< 0.001
BMI (kg/m^2^)	25.1 (3.7)	25.0 (3.7)	28.6 (4.4)	< 0.001
Heart rate (BPM)	67 (11)	67 (11)	71 (11)	0.008
Smoking	525 (30.7%)	504 (30.5%)	21 (38.2%)	0.22
Ischemic heart disease	82 (4.8%)	74 (4.5%)	8 (14.5%)	< 0.001
Heart failure	16 (0.9%)	16 (1.0%)	0 (0%)	0.46
Glucose (mmol/L)	5.5 (1.0)	5.5 (1.0)	6.2 (1.5)	< 0.001
HBA1c (%)	5.8 (0.5)	5.7 (0.5)	6.0 (0.5)	< 0.001
Triglycerides (mmol/L)	1.5 (0.8)	1.5 (0.8)	2.0 (1.2)	< 0.001
eGFR (mL/min)	76 (16)	76 (16)	76 (17)	0.94
Pro-BNP (pmol/L)	16, (8-30)	16 (7-30)	17 (11-30)	0.47
Total cholesterol (mmol/L)	5.5 (1.2)	5.5 (1.2)	5.7 (1.0)	0.30
Blood pressure lowering medication	222 (13.0%)	202 (12.2%)	20 (36.4%)	< 0.001
Lipid lowering medication	224 (13.1%)	207 (12.5%)	17 (30.9%)	< 0.001
Echocardiography				
LVEF (%)	59.7 (1.9)	59.7 (1.9)	59.9 (1.0)	0.61
LVIDd/height (cm/m)	2.8 (0.3)	2.8 (0.3)	2.8 (0.3)	0.52
LVMI (g/m^2^)	85.7 (22.1)	85.4 (21.9)	93.0 (24.4)	0.013
PWT (cm)	0.9 (0.2)	0.9 (0.2)	1.0 (0.2)	< 0.001
AWT (cm)	0.9 (0.2)	0.9 (0.2)	1.0 (0.2)	< 0.001
RWT (cm)	0.38 (0.08)	0.37 (0.08)	0.42 (0.11)	< 0.001
IVSD (cm)	1.0 (0.2)	1.0 (0.2)	1.1 (0.2)	< 0.001
LV concentric geometry	361 (21.1%)	338 (20.4%)	23 (41.8%)	< 0.001
LV hypertrophy categories				< 0.001
Normal geometry	1178 (69.1%)	1153 (69.9%)	25 (45.5%)	
Concentric remodeling	270 (15.8%)	253 (15.3%)	17 (30.9%)	
Eccentric hypertrohpy	166 (9.7%)	159 (9.6%)	7 (12.7%)	
Concentric hypertrohpy	91 (5.3%)	85 (5.2%)	6 (10.9%)	
LAVI (mL/m^2^)	19.4 (6.7)	19.4 (6.9)	20.2 (7.0)	0.44
E (m/s)	0.7 (0.2)	0.7 (0.2)	0.7 (0.2)	0.18
A (m/s)	0.7 (0.2)	0.7 (0.2)	0.8 (0.2)	< 0.001
E/A	1.2 (0.5)	1.2 (0.5)	0.9 (0.3)	< 0.001
E/e′	10.9 (4.7)	10.8 (4.6)	13.2 (4.9)	< 0.001
DT (ms)	165 (39)	165 (39)	177 (42)	0.026
s′ (cm/s)	5.9 (1.3)	6.0 (1.3)	5.7 (1.2)	0.16
e′ (cm/s)	7.5 (2.7)	7.6 (2.7)	5.9 (2.2)	< 0.001
a′ (cm/s)	6.4 (1.9)	6.3 (1.9)	7.1 (1.9)	0.008

*BP* blood pressure, *MAP* mean arterial pressure, *BMI* body mass index, *BPM* beats per minute, *AMI* acute myocardial infarction, *eGFR* estimated glomerular filtration rate, *BNP* brain natriuretic peptide, *LVEF* left ventricular ejection fraction, *GLS* global longitudinal strain, *LVIDd* left ventricular inner diameter at end diastole, *LVMI* left ventricular mass index, *PWT* posterior wall thickness, *AWT* average wall thickness, *RWT* relative wall thickness, *IVSD* interventricular septum diameter, *LAVI* left atrial volume index, *DT* deceleration time

**Table 2:** Baseline characteristics of participants stratified according to LV concentric geometry

Demographics	No LV concentric geometry	LV concentric geometry	p-value
N	1349 (78.9%)	361 (21.1%)	
Age (years)	54.6 (16.2)	64.8 (14.0)	< 0.001
Male	541 (40.1%)	135 (37.4%)	
Clinical characteristics			
Systolic BP (mmHg)	131.4 (22.1)	143.2 (21.2)	< 0.001
Diastolic BP (mmHg)	77.0 (11.5)	80.7 (13.6)	< 0.001
Hypertension	450 (33.4%)	215 (59.6%)	< 0.001
MAP (mmHg)	95.1 (13.6)	101.6 (14.0)	< 0.001
BMI (kg/m^2^)	25.0 (3.7)	25.7 (3.9)	0.001
Heart rate (BPM)	66 (11)	68 (12)	0.006
Smoking	406 (30.1%)	119 (33.0%)	0.29
Ischemic heart disease	59 (4.4%)	23 (6.4%)	0.11
Heart failure	11 (0.8%)	5 (1.4%)	0.32
Glucose (mmol/L)	5.4 (1.0)	5.7 (1.1)	< 0.001
HBA1c (%)	5.7 (0.5)	5.8 (0.5)	0.23
Triglycerides (mmol/L)	1.5 (0.8)	1.6 (0.9)	0.021
eGFR (mL/min)	77 (16)	74 (16)	< 0.001
Pro-BNP (pmol/L)	15 (7–28)	20 (9–37)	< 0.001
Total cholesterol (mmol/L)	5.5 (1.1)	5.8 (1.3)	< 0.001
Blood pressure lowering medication	139 (10.3%)	83 (23.0%)	< 0.001
Lipid lowering medication	151 (11.2%)	73 (20.2%)	< 0.001
Echocardiography			
LVEF (%)	59.7 (2.0)	59.8 (1.0)	0.24
LVIDd/height (cm/m)	2.9 (0.3)	2.6 (0.3)	< 0.001
LVMI (g/m^2^)	83.7 (20.8)	93.0 (25.0)	< 0.001
PWT (cm)	0.8 (0.1)	1.1 (0.1)	< 0.001
AWT (cm)	0.9 (0.1)	1.1 (0.2)	< 0.001
RWT (cm)	0.34 (0.05)	0.49 (0.07)	< 0.001
IVSD (cm)	0.9 (0.2)	1.1 (0.2)	< 0.001
LV hypertrophy categories			N/A
Normal geometry	1178 (87.6%)	0	
Concentric remodeling	0	270 (74.8%)	
Eccentric hypertrohpy	166 (12.4%)	0	
Concentric hypertrohpy	0	91 (25.2%)	
LAVI (mL/m^2^)	19.4 (6.7)	19.6 (7.6)	0.56
E (m/s)	0.7 (0.2)	0.7 (0.2)	< 0.001
A (m/s)	0.7 (0.2)	0.7 (0.2)	< 0.001
E/A	1.2 (0.5)	1.0 (0.4)	< 0.001
E/e′	10.4 (4.2)	12.8 (5.9)	< 0.001
DT (ms)	163 (36)	174 (49)	< 0.001
s′ (cm/s)	6.0 (1.3)	5.7 (1.3)	< 0.001
e′ (cm/s)	7.8 (2.7)	6.2 (2.3)	< 0.001
a′ (cm/s)	6.2 (1.9)	6.9 (1.8)	< 0.001

*BP* blood pressure, *MAP* mean arterial pressure, *BMI* body mass index, *BPM* beats per minute, *AMI* acute myocardial infarction, *eGFR* estimated glomerular filtration rate, *BNP* brain natriuretic peptide, *LVEF* left ventricular ejection fraction, *GLS* global longitudinal strain, *LVIDd* left ventricular inner diameter at end diastole, *LVMI* left ventricular mass index, *PWT* posterior wall thickness, *AWT* average wall thickness, *RWT* relative wall thickness, *IVSD* interventricular septum diameter, *LAVI* left atrial volume index, *DT* deceleration time

## Results

### Population, outcome and follow-up

Median follow-up time was 12.6 years (IQR: 12.0–12.8 years). Follow-up was a 100%. End-point was incident DM. A total of 55 participants (3.3%) developed DM during follow-up.

### Baseline characteristics of the population stratified per DM at follow-up

Participants who developed DM were significantly older and displayed higher values of systolic blood pressure (BP), diastolic BP and mean arterial pressure (Table 1). Also, they displayed higher values of body mass index and heart rate, and the prevalence of IHD was higher among participants who developed DM (Table 1). Furthermore, they displayed significantly higher values of blood glucose, HbA1c levels and blood triglycerides (Table 1).

Participants who developed DM displayed higher values of LVMI, PWT, average wall thickness, RWT, IVSD, A, E/e′, DT and a′ (Table 1). Individuals who developed DM displayed significantly lower values of E/A, s′ and e′ (Table 1). The prevalence of LV concentric remodelling, LV concentric hypertrophy and LV concentric geometry was significantly higher in participants who developed DM during follow-up (Table 1). Notably, at baseline, the prevalence of LV concentric geometry was twice as high (20% vs 42%, p < 0.001) in individuals who developed DM during follow-up.

### Baseline characteristics stratified per LV concentric geometry

Participants with concentric LV geometry were characterized by higher blood pressure, higher body mass index and higher heart rate (Table 2). Blood levels of glucose, triglycerides, total cholesterol and pro-BNP were also significantly higher in participants with concentric LV geometry (Table 2). They also had significantly higher LVMI, PWT, average wall thickness, RWT, IVSD, A, E/e′, DT and a′ (Table 2). Finally, they displayed significantly lower values of LVIDd/height, E, E/A, s′ and e′ (Table 2).

### Prediction of incident DM in the general population

In univariable Cox regression, LV concentric geometry, LVMI, IVSD, PWT, average wall thickness, RWT, A, E/A E/e′, DT, e′ and a′ were significant predictors of incident DM (Table 3). In univariable Cox regression, individuals with LV concentric geometry exhibited an approx. 3 times greater risk of developing DM compared to participants without LV concentric geometry (Fig. 2). Both LV concentric remodelling and LV concentric hypertrophy were associated with an increased risk of developing DM, and no statistically significant difference in risk was present between the two groups (LV concentric remodelling: HR 3.22, 95% CI 1.74–5.98, p < 0.001) (LV concentric hypertrophy: HR 3.61, 95% CI 1.48-8.80, p = 0.005) (p for difference: p = 0.81). In univariable Cox regression interaction analysis, the association between LV concentric geometry or RWT and DM was not significantly modified by sex (LV concentric geometry, p for interaction: p = 0.19) (RWT, p for interaction: p = 0.88). Additionally, the risk of developing DM during follow-up increased exponentially with increasing degree of LV concentricity evaluated as RWT (Figs. 3 and 4).

**Table 3:** Echocardiographic predictors of incident DM (n = 55) using Cox regression

Unadjusted	Hazard ratio	p-value
LV concentric geometry	HR 2.92, 95% CI 1.71–5.00	< 0.001
LVIDD/height (per 1 cm/m increase)	HR 0.73, 95% CI 0.0.29–1.87	0.51
LVMI (per 5 g/m^2^ increase)	HR 1.08, 95% CI 1.02–1.13	0.004
IVSD (per 1 mm increase)	HR 1.31, 95% CI 1.18–1.46	< 0.001
PWT (per 1 mm increase)	HR 1.35, 95% CI 1.20–1.52	< 0.001
AWT (per 1 mm increase)	HR 1.49, 95% CI 1.30–1.71	< 0.001
RWT (per 0.1 increase)	HR 1.71, 95% CI 1.38–2.12	< 0.001
LAVI (per 1 mL/m^2^ increase)	HR 1.02, 95% CI 0.99–1.06	0.20
E (per 10 cm/s decrease)	HR 1.13, 95% CI 0.95–1.34	0.17
A (per 10 cm/s increase)	HR 1.50, 95% CI 1.33–1.70	< 0.001
E/A ratio (per 0.1 decrease)	HR 1.27, 95% CI 1.15–1.40	< 0.001
E/e′ ratio (per 1 increase)	HR 1.09, 95% CI 1.05–1.13	< 0.001
DT (per 10 ms increase)	HR 1.08, 95% CI 1.02–1.14	0.009
s′ (per 1 cm/s increase)	HR 0.81, 95% CI 0.64–1.02	0.07
e′ (per 1 cm/s increase)	HR 0.76, 95% CI 0.67–0.85	< 0.001
a′ (per 1 cm/s increase)	HR 1.22, 95% CI 1.05–1.40	0.008

Model 1	Hazard ratio	p-value
A (per 10 cm/s increase)	HR 1.22, 95% CI 1.02–1.46	0.028
LV Concentric Geometry	HR 1.92, 95% CI 1.09–3.38	0.023
RWT (per 0.1 increase)	HR 1.43, 95% CI 1.09–1.87	0.009

Model 2	Hazard ratio	p-value
LV Concentric Geometry	HR 1.99, 95% CI 1.11–3.57	0.021
RWT (per 0.1 increase)	HR 1.41, 95% CI 1.06–1.86	0.017

Model 1 is adjusted for age, sex, hypertension, smoking status, total cholesterol levels, triglyceride levels, BMI, blood glucose, HbA1C levels, pro-BNP, prevalent ischemic heart disease and prevalent heart failure. Model 2 is adjusted for the same variables as Model 1 with the addition of A. In the multivariable models, only parameters reaching statistical significance are shown

*LVIDD/height* left ventricular internal diameter at end diastole indexed to height, *LVMI* left ventricular mass index, *IVSD* interventricular septum diameter, *PWT* posterior wall thickness, *AWT* average wall thickness, *RWT* relative wall thickness, *LAVI* left atrial volume index, *DT* deceleration time

In a multivariable model (Model 1, Table 3) adjusting for age, sex, hypertension, smoking status, total cholesterol levels, blood triglycerides, BMI, blood glucose, HbA1c levels, pro-BNP, prevalent IHD and prevalent HF, only A, RWT and LV concentric geometry remained independent predictors of DM (Table 3). In a final multivariable model (Model 2, Table 3) adjusting for the same variables as Model 1 with further adjustment for A, only RWT and LV concentric geometry remained independent predictors of DM (Table 3). Additional adjustment of our final multivariable model for either blood pressure lowering or lipid lowering medication did not attenuate the prognostic value of RWT and LV concentric geometry (Lipid lowering: Concentric geometry HR 1.83, 95%CI 1.01–3.30, p = 0.046; RWT HR 1.35, 95%CI 1.02–1.78, per 0.1 increase, p = 0.037) (blood pressure lowering: concentric geometry HR 1.98, 95%CI 1.10–3.56, p = 0.022; RWT HR 1.43, 95%CI 1.07–1.90, per 0.1 increase, p = 0.014). The results were similar in competing risk regression treating death as a competing event (Additional file 1: Table S1).

### Association of RWT with diastolic function

In restricted cubic spline regression LV concentricity as evaluated by RWT was significantly associated with diastolic function (Fig. 5). Diastolic function evaluated as e′ and E/e′ declined with increasing degree of LV concentricity (Fig. 5).

### Incremental prognostic value in addition to established risk factors for development of DM

We assessed the incremental prognostic value of myocardial indices derived from echocardiography when added to already established risk factors for DM (age, sex, total cholesterol, triglycerides, haemoglobin A1C levels, systolic blood pressure, and BMI). In reclassification analysis, only RWT and LV concentric geometry provided incremental prognostic value when added to established risk factors (LV concentric geometry: Continuous NRI 0.429, 95% CI 0.023–0.680; IDI 0.010, 95%CI 0.000–0.033) (RWT: continuous NRI 0.431, 95% CI 0.040–0.760; IDI 0.008, 95%CI 0.000–0.037).

## Discussion

In this general population study, we demonstrate that measures of LV structure derived from an echocardiographic examination independently predict the development of DM 10 years following examination. Specifically, we demonstrate that a LV concentric geometric pattern is an independent predictor of DM in the general population, even after adjustment for established risk factors for DM. Furthermore, we show that the risk of incident DM rises continuously with increasing degree of LV concentricity as evaluated by RWT. Finally, we show that this prognostic information is incremental to established predictors of DM, indicative of a strong association between LV concentric geometry and DM.

### Echocardiography, DM and diabetic cardiomyopathy

The notion of a true and distinct diabetic cardiomyopathy has been around for a long time, dating all the way back to the 1950s when Lundbæk, an internist in Denmark, observed frequent myocardial dysfunction in elderly patients with DM [23]. He suggested that DM patients may have heart disease in the absence of coronary blockage [23]. He became the first to suggest the existence of a specific diabetic form of cardiomyopathy. Now, two distinct phenotypes of diabetic cardiomyopathy have been suggested: an eccentric, dilated form with reduced systolic function (HFrEF phenotype), and a restricted, concentric form characterized by diastolic dysfunction (HFpEF phenotype) [13]. The HFpEF phenotype is the one most often encountered by clinicians [13]. Several cross-sectional studies have demonstrated impaired diastolic function and increased LV mass in DM individuals [4, 5, 7, 24]. In our study, diastolic parameters (A, DT, E/A, E/e′, e′, a′) were impaired and LVMI was increased in participants who developed DM during follow-up, supporting the findings of previous studies. However, only peak A-wave remained significant after adjustment for clinical characteristics, suggesting that the prognostic value of the other diastolic parameters was secondary to associations with clinical characteristics. In early diastolic dysfunction and with increasing age along with subtle wall hypertrophy, the A wave increases in magnitude due to increased atrial contribution to LV filling to compensate for the decreased inflow during early diastole (E wave), often referred to as “atrial kick” [25]. However, peak A-wave did not remain significant in a final model adjusting for clinical characteristics and LV concentric geometry. In this model, only LV concentric geometry and RWT were independent predictors of outcome, suggesting that the significance of A was secondary to clinical characteristics and the degree of LV concentricity. It is widely recognized that cardiac concentric remodelling can contribute to diastolic dysfunction, and thus our results support the hypothesis that diastolic parameters are associated with DM secondary to LV concentricity. A recent study by our group, examining cardiac function in a population of DM individuals, found that individuals with DM were mainly characterized by increased LV concentricity evaluated as RWT due to increased LV wall thickness and smaller LV cavities [7]. In the same study, we found that the severity of LV concentricity evaluated by RWT correlated significantly to the duration of DM [7]. Similar relationships between DM and LV concentricity have been found by other authors: In a recent cross-sectional study of type 2 DM patients, De Jong et al. [26] reported that LV concentricity and RWT were significantly increased in both obese and non-obese type 2 DM patients as compared to metabolically healthy obese patients, even in the absence of hypertension. Roberts et al. [27] compared the exercise capacity of type 2 DM patients to that of healthy age and sex-matched controls and found that increased sedentary behaviour and reduced LVIDd were significantly associated with reduced exercise capacity in patients with type 2 DM (in addition, type 2 DM patients also displayed increased RWT as compared to healthy controls). These findings concur with our results, since LV concentric geometry and RWT were the only echocardiographic predictors of incident DM in the present study, and since the risk of DM increased continuously with increasing degree of LV concentricity as evaluated by RWT, suggesting that LV concentricity is indeed correlated to abnormal glucose metabolism.

The finding that the prognostic value of diastolic parameters is secondary to indices of LV concentricity is supported by the suspected pathophysiological mechanisms underlying diabetic cardiomyopathy. It is thought, in the HFpEF phenotype, that hyperglycaemia, lipotoxicity and insulin resistance cause cardiomyocyte hypertrophy and increased resting tension, along with collagen deposition between cells [13]. This leads to LV wall thickening with a concomitant increase in stiffness and decrease in diastolic function. These pathophysiological considerations are reflected in our results, since participants with concentric LV geometry had significantly higher levels of blood glucose, blood triglycerides and cholesterol levels. They also had significantly higher body mass index and blood pressure and lower estimated glomerular filtration rates. These are all risk factors for CVD [28–31], and they contribute significantly to the increased risk of CVD seen in DM [32]. Thus, the degree of LV concentricity appears to scale with the degree of dyslipidaemia, hyperglycaemia, hypertension, and body mass index even before development of DM, supporting its prognostic value in predicting DM.

### Target organ damage and prediction of DM

Conventionally, it has been thought that a clear temporal relationship existed between cardiovascular risk factors and the development of end organ damage such as LV diastolic dysfunction, LV hypertrophy and LV systolic impairment. However, it has recently been shown that LV hypertrophy and arterial stiffness, markers of end organ damage, are predictors of incident hypertension in both normotensive and prehypertensive individuals [33, 34]. This raises questions about the temporal relationship between cardiovascular risk factors and end organ damage, indicating that end organ damage may contribute with novel prognostic value. Besides our study, one study has previously investigated the prognostic role of echocardiography with respect to development of DM in the general population. In this study, Park and Colleagues investigated the prognostic value of echocardiography in predicting incident type 2 DM in a cohort of 1817 non-diabetic individuals from Korea [9]. The age of their population was lower (mean 54 years vs mean 57.9 years) and the follow-up was shorter (6 years vs median 12.6 years) when compared to our study. Also, the prevalence of hypertension in their sample was much lower (22.1% vs 38.7%) than in our sample indicative of a younger and healthier population. During follow-up 273 individuals (15%) developed type 2 DM, which is higher than what we found in our study. This may be explained by the lower age of their study sample. Due to the higher age of participants in our study many had already developed DM at baseline and were therefore excluded. Park and Colleagues found that markers of diastolic dysfunction (e′ and E/e′) predicted incident DM. After adjustment for clinical risk factors, only e’ and the presence of diastolic dysfunction (yes/no) were independent predictors of DM. No results regarding the prognostic value of LV concentric geometry or RWT in predicting DM in the final multivariable model are shown, however, RWT was significantly higher in individuals who developed type 2 DM during follow-up (0.39 SD 0.07 vs 0.36 SD 0.06). This suggests that LV concentric geometry may have been an independent predictor of DM in their study as well, however no results regarding LV concentric geometry was reported. Nevertheless, it is possible that the prognostic value of echocardiography differs between ethnicities with regards to prediction of DM. Hence more research is needed to explain the differences between our two studies. However, altered LV concentricity seems to correspond more closely to the pathophysiological mechanisms suspected to underlie the cardiac alterations seen in the HFpEF phenotype of diabetic cardiomyopathy, and our results suggest that the predictive value of many diastolic parameters in predicting DM is secondary to LV concentric geometry. To our knowledge, ours is the second study to evaluate the prognostic value of echocardiography in predicting development of DM in the general population, and therefore, our results should be viewed as exploratory and hypothesis generating. More research, by independent groups in similar populations, is needed before any significant conclusions can be drawn. In summary, we show that end organ damage can contribute with prognostic value in predicting DM in the general population, and that this organ damage precedes the development of DM.

### Limitations and future considerations

Several limitations to this study must be acknowledged. Firstly, mean age at baseline of participants included into this study was 56.6 years (SD: 16.3 years), and thus the prevalence of DM before exclusion of diabetic individuals was 10.9% (241 individuals). These were all excluded from the study. Therefore, due to the high age of this general population sample, a large proportion had already developed DM at baseline and were therefore excluded. Furthermore, we assessed the development of DM by ICD-10 codes, and therefore we do not know how rigorously or by what methods participants have been monitored for the development of DM. Due to unknown differences in the rigour of monitoring of participants, the time from echocardiographic examination to DM diagnosis may have been overestimated, since participants may not have sought out medical consultation at the onset of symptoms from DM. This could potentially alter the temporal relationship between RWT values and DM development observed in this study. Furthermore, in the Copenhagen City Heart study, prevalent DM was defined using the old HbA1c cut-off value (HbA1c ≥ 7.0%). Today a cut-off value of HBA1c ≥ 6.5% is used [35]. Therefore, our definition of prevalent DM may have underestimated the true prevalence. Also, the general population in Denmark is mainly of Caucasian ethnicity, and therefore we cannot extrapolate our results to other races—stressing this is particularly important when considering ethnicity in itself is a significant risk factor for DM, and that ethnicity modifies the contributions of many other risk factors for cardiovascular disease in diabetic individuals [11, 12]. We did not have information on the prevalence of rare cardiomyopathies such as hypertrophic cardiomyopathy, which may affect LV geometry. However, undiagnosed cases of rare cardiomyopathies are unlikely to have affected our results given their very low prevalence in the general population. Also, since rare cardiomyopathies do not cause DM, potential inclusion of such patients would only serve to weaken our results. Yet, we still found significant associations between LV concentric geometry and DM. A final limitation is related to the method of outcome assessment in the present study. In this study, DM outcomes were assessed through the Danish National Board of Health’s National Patient Registry using ICD 10 codes. However, this means that receiving a diagnosis of DM necessitates some type of hospital contact. This hospital contact can either be directly related to DM, or it can be unrelated to the presence of DM and the presence of DM may then be discovered through patient history or examination/assessment. Given the long follow-up of our study (median 12.6 years) and the high age of participants at baseline (mean 57 years) it is reasonable to assume that most patients will have at least one hospital contact not related to a potential DM diagnosis facilitating the discovery of DM if present. Yet, this does constitute a limitation of the present study and could have affected our results. In addition, it is also possible that the DM outcomes detected may represent sicker DM patients possibly requiring hospitalisation at DM debut since outcome assessment is related to hospital contact. Thus, although our study provides important hypothesis generating results, ideally, our findings should be evaluated in multiple ethnicities, in a younger study population and using a study design including closer, dedicated outcome assessment.

## Conclusion

Altered LV geometry may precede the development of DM. LV concentric geometry determined by echocardiography and the severity of LV concentricity evaluated as RWT are independent predictors of incident DM in the general population.

## Additional file

**Additional file 1: Table S1.** Competing risk regression with prediction of Diabetes Mellitus (n=55) while treating death (n=313) as a competing event.

## Abbreviations

LV: left ventricular
DM: diabetes mellitus
RWT: relative wall thickness
LVIDd: left ventricular inner diastolic diameter
IQR: interquartile range
BMI: body mass index
HR: hazard ratio
CVD: cardiovascvular disease
BNP: brain natriuretic peptide
IHD: ischemic heart disease
TDI: tissue Doppler imaging
WMSI: wall motion score index
LVEF: left ventricular ejection fraction
LVMI: left ventricular mass index
BSA: body surface area
PWT: posterior wall thickness
IVSD: interventricular septum diameter at end diastole
AIC: Akaike Information Criterion
DT: deceleration time
NRI: net reclassification index
HFrEF: heart failure with reduced ejection fraction
HFpEF: heart failure with preserved ejection fraction
