# Supplementary material for: Left ventricular concentric geometry predicts incident diabetes mellitus independent of established risk factors in the general population: the Copenhagen City Heart Study
Source: Cardiovasc Diabetol. 2019 Mar 20;18:37. doi: 10.1186/s12933-019-0842-0 (PMC6427881; doi:10.1186/s12933-019-0842-0)
Supplement: Supplementary file 1 — Additional file 1: Table S1. Competing risk regression with prediction of Diabetes Mellitus (n=55) while treating death (n=313) as a competing event. [file 12933_2019_842_MOESM1_ESM.docx]

Concentric Left Ventricular Geometry predicts Incident Diabetes Mellitus Independent of Established Risk Factors in the General Population: The Copenhagen City Heart Study

**Authors:** Daniel Modin, MB; Rasmus Mogelvang, MD, PhD; Peter Godsk Jørgensen, MD, PhD; Magnus Thorsten Jensen, MD, PhD; Jelena P. Seferovic MD, PhD; Tor Biering-Sørensen, MD, PhD

Additional Material

**Table S1**

Competing risk regression with prediction of Diabetes Mellitus (n=55) while treating death (n=313) as a competing event.

|  |  |  |  |
| --- | --- | --- | --- |
| **Unadjusted** | Subdistribution Hazard Ratio | P-value |  |
| LV Concentric geometry | SHR 2.70, 95CI 1.59-4.62 | <0.001 |  |
| LVIDD/Height (per 1cm/m increase) | SHR 0.76, 95CI 0.26-2.24 | 0.62 |  |
| LVMI (per 5g/m^2^ increase) | SHR 1.07, 95CI 1.02-1.12 | 0.005 |  |
| IVSD (per 1mm increase) | SHR 1.27, 95CI 1.15-1nB.39 | <0.001 |  |
| PWT (per 1mm increase) | SHR 1.34, 95CI 1.22-1.47 | <0.001 |  |
| RWT (per 0.1 increase) | SHR 1.66, 95CI 1.38-1.99 | <0.001 |  |
| LAVI (per 1ml/m^2^ increase) | SHR 1.02, 95CI 0.98-1.05 | 0.32 |  |
| E (per 10 cm/s decrease) | SHR 1.11, 95CI 0.95-1.30 | 0.19 |  |
| A (per 10 cm/s increase) | SHR 1.45, 95CI 1.29-1.63 | <0.001 |  |
| E/A ratio (per 0.1 decrease) | SHR 1.24, 95CI 1.13-1.35 | <0.001 |  |
| E/e’ ratio (per 1 increase) | SHR 1.08, 95CI 1.04-1.11 | <0.001 |  |
| DT (per 10 ms increase) | SHR 1.07, 95CI 1.02-1.12 | 0.010 |  |
| s’ (per 1 cm/s increase) | SHR 0.85, 95CI 0.68-1.06 | 0.15 |  |
| e’ (per 1 cm/s increase) | SHR 0.78, 95CI 0.70-0.87 | <0.001 |  |
| a’ (per 1 cm/s increase) | SHR 1.21, 95CI 1.05-1.39 | 0.010 |  |
| **Model 1** | Subdistribution Hazard Ratio | P-value |  |
| A (per 10 cm/s increase) | SHR 1.21, 95CI 0.99-1.49 | 0.07 |  |
| LV Concentric Geometry | SHR 1.97, 95CI 1.13-3.42 | 0.016 |  |
| RWT (per 0.1 increase) | SHR 1.43, 95CI 1.09-1.89 | 0.011 |  |
| **Model 2** | Subdistribution Hazard Ratio | P-value |  |
| LV Concentric Geometry | SHR 2.12, 95CI 1.18-3.83 | 0.012 |  |
| RWT (per 0.1 increase) | SHR 1.48, 95CI 1.10-1.98 | 0.009 |  |
|  |  |  |  |

*Model 1 is adjusted for age, sex, hypertension, smoking status, total cholesterol levels, triglyceride levels, BMI, blood glucose, HbA1C levels, pro-BNP, prevalent ischemic heart disease and prevalent heart faiulure. Model 2 is adjusted for the same variables as Model 1 with the addition of A. In the multivariable models, only parameters reaching statistical significance are shown. LVIDD/Height, left ventricular internal diameter at end diastole indexed to height; LVMI, left ventricular mass index; IVSD interventricular septum diameter; PWT, posterior wall thickness; RWT, relative wall thickness; LAVI left atrial volume index; DT, deceleration time.*
